# Frequency of health care utilization in the year prior to completed suicide: A Danish nationwide matched comparative study

**DOI:** 10.1371/journal.pone.0214605

**Published:** 2019-03-27

**Authors:** Henrik Schou Pedersen, Morten Fenger-Grøn, Bodil Hammer Bech, Annette Erlangsen, Mogens Vestergaard

**Affiliations:** 1 Research Unit for General Practice, Aarhus University, Aarhus, Denmark; 2 Department of Public Health, Aarhus University, Aarhus, Denmark; 3 Danish Research Institute for Suicide Prevention, Mental Health Centre Copenhagen, Copenhagen, Denmark; 4 Center of Mental Health Research, Australian National University, Canberra, Australia; 5 Department of Mental Health, Johns Hopkins Bloomberg School of Public Health, Baltimore, United States of America; University of Toronto, CANADA

## Abstract

**Background:**

Suicide accounts for more than 800,000 annual deaths worldwide. Some of these deaths may be preventable by timely identification of individuals at risk and effective intervention. General practitioners (GPs) may have the potential to play an important role in this process.

**Aim:**

The present study aimed to assess the frequency of primary health care utilization in the year preceding suicide.

**Methods:**

Using Danish national registers, we identified all persons who died by suicide in Denmark from 1997 through 2013 and assessed the frequency of their primary care utilization and compared it with that of an age- and sex-matched reference group sampled from the background population.

**Results:**

We identified 11,191 persons who died by suicide (males: 8,095, females: 3,096). Compared with the reference group (N = 55,955), a greater proportion attended general practice in the year before index date (83% vs. 76%). In the last month before index date, these figures were 32.0% and 19.4%, respectively, corresponding to a difference of 12.0 95% CI: (11.1; 12.9) percentage points after adjustment for demographic characteristics and physical comorbidity. Suicide cases had a higher GP attendance in every week in the year before suicide, but the difference increased specifically in the last four months.

**Conclusion:**

More than 30% attended the GP in the month before the suicide. This indicates that general practice could be a possible place to identify suicide cases and offer intervention. However, although this proportion represents a markedly higher GP attendance than seen in the reference group, almost 70% of those who died by suicide did not attend primary care in the month before the suicide. Our study suggests that it is important that the GPs have easy access to effective suicide prevention programs for patients at risk of suicide, and that persons with suicidal thoughts are encouraged to contact their GP.

## Introduction

More than 800,000 persons die by suicide every year [1]. Mounting evidence suggests that some suicides could be prevented if persons at risk are identified and offered specialized treatment [2–4].

About 98% of the population in Denmark is listed with a specific general practice and receives publicly funded health care services free of charge [5]. General practitioners (GPs) provide first-contact care to unselected health problems and act as gatekeepers to secondary care. Several studies have found that a large proportion of the persons who die by suicide have contacted the GP in the year leading up to the suicide [6–13]. However, previous studies have been limited by small sample sizes [8–12,14], lack of reference group [6,7,9–12], potentially incomplete data [7], and restrictions to selected groups [8,10]. All these limitations challenge the generalizability of findings, including assessment of the extent to which the GP utilization among persons who die by suicide differ from that of persons with similar characteristics who do not die by suicide.

In a large nationwide matched comparative study based on all recorded suicides in Denmark from 1997 through 2013, we aimed to add volume to the body of work on the proportion of individuals attending primary care on a weekly basis in the year prior to suicide and further to compare this proportion with that of a matched reference group in regression analyses adjusted for age, sex, calendar time, socioeconomic characteristics, and physical comorbidity to see if they could be identified in general practice.

## Methods

We conducted a matched comparative study based on information from multiple Danish national registers. Using the unique personal identification number assigned to all Danish citizens at birth or immigration [15], we linked information at the individual level across the registers. We assessed the frequency of daytime face-to-face consultations provided by the GP in the year preceding suicide in persons who died by suicide from 1 January 1997 through 31 December 2013 and compared their frequency of utilization to that of the background population adjusted for important confounding variables [16]. To obtain a more detailed picture of the frequency of health care utilization, we explored different types of services provided by the GP, medicine redemptions, and contacts with the psychiatric treatment system in secondary care.

### Participants

Using the Danish Register of Causes of Death [17], we identified all individuals recorded with suicide from 1 January 1997 through 31 December 2013. Deaths by suicide were identified using codes X60-X84 and Y87 of the International Classification of Diseases, 10^th^ revision (ICD-10). For each suicide case, we randomly sampled five reference persons from the background population matched on sex and age (+/- one month) who were still alive and listed as living in Denmark at the date of the suicide (index date). Information on sex and age was obtained from the Danish civil registration system (CRS) [18], and information on migration was provided by Statistics Denmark. Only persons who had been living in Denmark for at least 12 months prior to the index date were eligible to participate in the study. Sampling was done without replacement for each suicide case and with replacement between suicide cases, i.e. each person could serve as a reference for more than one suicide case, but only once for the same suicide case. Reference persons could later be included as a suicide case if they died by suicide.

### Measurements

We measured the utilization of daytime face-to-face consultations identified in the National Health Insurance Service Register (NHISR) [5]. The NHISR contains data on type of all contacts to the GP, and all data are based on the GPs’ invoices for provided services. We did not have access to the actual treatment date, but we were able to identify the week of the remuneration of the GP and the corresponding number of contacts in this week. To obtain a more general picture on the frequency of health care utilization, we investigated the usage of nine different types of services in general practice in the year prior to index date: out-of-hours (OOH) face-to-face consultations, talk therapy sessions, electrocardiograms, blood sample tests, hemoglobin measuring tests, rapid strep tests, urine sample tests, spirometer tests, and C-reactive protein tests (Table B in S1 Appendix). Furthermore, data on redeemed prescriptions were obtained from the Danish Register of Medicinal Product Statistics [19]. We investigated prescription redemptions categorized according to the Anatomical Therapeutic Chemical (ATC) classification system for antipsychotics (ATC: N05A) and antidepressants (ATC: N06A). As the duration of a prescription could span many months, and a treatment regime could comprise multiple prescription redemptions, we assumed that each prescription lasted for four months, and a person redeeming a new prescription was considered to be under treatment for another four months. In addition, information on contacts with the psychiatric treatment system in secondary care was obtained from the Danish Psychiatric Central Research Register (PCR) [20].

### Covariates

Thirty-one different somatic diseases were identified and included in the analyses as dichotomous variables (Table F in S1 Appendix). The diseases were defined according to an algorithm developed by Prior et al. [21] encompassing 39 different somatic and mental diseases treated in primary or secondary care. These were identified through the Danish National Patient Registry [22], the PCR, the Danish Cancer Registry [23], the Danish Register of Medicinal Product Statistics, the NHISR, and the National Diabetes Register [24].

Sex and age at index date was found in the CRS.

Level of highest attained education and cohabitation status were obtained from annually updated information from Statistics Denmark, which also provided us with the partner’s personal identification number. If a participant’s partner had died within the last year, this participant was categorized as recently bereaved. Date of death was identified in the CRS. Cohabitation status was divided into four groups (single, recently bereaved, cohabiting, married). Educational level was divided into three groups according to years of schooling (≤10 years, 11–15 years, ≥16 years) [25]. Persons with no information on highest attained educational level were assigned to the lowest category.

All variables, except sex, age at index date, and calendar time at index date, were considered to be time-dependent and updated continuously.

### Statistical analysis

We dichotomized all contacts and types of services into “usage” and “non-usage” for the time unit of interest. These Bernoulli-distributed variables were analyzed using a generalized linear model with identity link and with varying extent of adjustment. This approach yielded proportion differences (PDs) as the measure of association. Cluster robust variance estimation was applied on the person level to account for the fact that persons could be included in the dataset more than once [26]. Confidence intervals (CI) were calculated on the 95% level.

Regression models were analyzed using three different a priori chosen adjustment models. Model 1 contained the matching variables with an interaction between age and sex. To allow non-linear associations we modelled the continuous variables in a flexible manner using restricted cubic splines with a pre-specified number of knots [27]. We placed knots at equally spaced percentiles to ensure a good fit. We modelled calendar-time and sex-specific splines using four knots. In the two other models, we aimed to see whether socioeconomic status (model 2) and presence of somatic comorbid diseases (model 3) explained some of the observed difference. Besides the variables from model 1, model 2 included educational level, cohabitation status, and the interaction between these variables and age. Model 3 included model 2 and dummy variables for all 31 somatic diseases.

Daytime face-to-face attendance were analyzed in both the entire year before index date and in the last month before index date. For the year prior to the index date, we included number of weeks before index date as both a continuous variable modelled by five-knotted restricted cubic splines and as a categorical variable for illustrative purposes. For all other types of contacts, we investigated frequency of utilization in the year before index date with number of months before index date included as a categorical variable.

In one sensitivity analysis, we excluded participants listed with a GP who requested remuneration for less than 35 weeks in a calendar year since this could be a sign that the GP did not request remuneration systematically every week, which could challenge the precision of the calculation of the treatment week. The algorithm used to identify GP affiliation was defined and developed by Kjærsgaard et al. [28]. In another sensitivity analysis, we excluded participants who had been hospitalized at some time point in the year before index date and, therefore, were unable to attend the GP. We identified all hospitalizations in the Danish National Patient Registry and in the PCR.

All analyses were performed using Stata 13, StatCorp LP, College Station, Texas, USA.

### Ethics statement

The study was approved by the Danish Data Protection Agency, the Danish Health Data Authority, and Statistics Denmark. This type of study did not need approval from the regional ethics committee as all patient information was based on register data, which were anonymized and de-identified prior to analysis by Statistics Denmark.

## Results

We identified 11,191 persons (males: 8,095; females: 3,096) who died by suicide and 55,955 references matched on age and sex. The annual number of suicides decreased slightly over the study period. The number of suicides increased with increasing age until age 60 and decreased after age 60. Compared to the reference group, persons dying by suicide were more likely to have co-existing somatic diseases, have a short education, be single, and to have recently lost a partner (Table 1).

**Table 1:** Characteristics of the cohort one month before index date.

	Reference group	Suicide group	Total
	N (column %)	N (column %)	N (column %)
**Total**	55,955 (100%)	11,191 (100%)	67,146 (100%)
**Sex**			
Male	40,475 (72.3%)	8,095 (72.3%)	48,570 (72.3%)
Female	15,480 (27.7%)	3,096 (27.7%)	18,576 (27.7%)
**Age (years)**			
-20	1,345 (2.4%)	269 (2.4%)	1,614 (2.4%)
21–30	4,480 (8%)	896 (8%)	5,376 (8%)
31–40	7,305 (13.1%)	1,461 (13.1%)	8,766 (13.1%)
41–50	10,925 (19.5%)	2,185 (19.5%)	13,110 (19.5%)
51–60	11,210 (20%)	2,242 (20%)	13,452 (20%)
61–70	8,365 (14.9%)	1,673 (14.9%)	10,038 (14.9%)
71–80	6,790 (12.1%)	1,358 (12.1%)	8,148 (12.1%)
81–90	4,645 (8.3%)	929 (8.3%)	5,574 (8.3%)
91-	890 (1.6%)	178 (1.6%)	1,068 (1.6%)
**Year of index date**			
1997	4,045 (7.2%)	809 (7.2%)	4,854 (7.2%)
1998	3,720 (6.6%)	744 (6.6%)	4,464 (6.6%)
1999	3,765 (6.7%)	753 (6.7%)	4,518 (6.7%)
2000	3,605 (6.4%)	721 (6.4%)	4,326 (6.4%)
2001	3,615 (6.5%)	723 (6.5%)	4,338 (6.5%)
2002	3,410 (6.1%)	682 (6.1%)	4,092 (6.1%)
2003	3,120 (5.6%)	624 (5.6%)	3,744 (5.6%)
2004	3,240 (5.8%)	648 (5.8%)	3,888 (5.8%)
2005	3,125 (5.6%)	625 (5.6%)	3,750 (5.6%)
2006	3,235 (5.8%)	647 (5.8%)	3,882 (5.8%)
2007	2,930 (5.2%)	586 (5.2%)	3,516 (5.2%)
2008	3,020 (5.4%)	604 (5.4%)	3,624 (5.4%)
2009	3,090 (5.5%)	618 (5.5%)	3,708 (5.5%)
2010	2,815 (5%)	563 (5%)	3,378 (5%)
2011	2,950 (5.3%)	590 (5.3%)	3,540 (5.3%)
2012	3,260 (5.8%)	652 (5.8%)	3,912 (5.8%)
2013	3,010 (5.4%)	602 (5.4%)	3,612 (5.4%)
**Number of comorbid somatic diseases**			
0	33,270 (59.5%)	5,795 (51.8%)	39,065 (58.2%)
1	10,752 (19.2%)	2,398 (21.4%)	13,150 (19.6%)
≥2	11,933 (21.3%)	2,998 (26.8%)	14,931 (22.2%)
**Educational level**			
≤10	22,517 (40.2%)	5,434 (48.6%)	27,951 (41.6%)
11–15	24,387 (43.6%)	4,416 (39.5%)	28,803 (42.9%)
≥16	9,051 (16.2%)	1,341 (12%)	10,392 (15.5%)
**Cohabitation status**			
Single	18,447 (33%)	6,056 (54.1%)	24,503 (36.5%)
Recently bereaved	361 (0.6%)	211 (1.9%)	572 (0.9%)
Cohabiting	6,209 (11.1%)	977 (8.7%)	7,186 (10.7%)
Married	30,938(55.3%)	3,947(35.3%)	34,885 (60%)

**Fig 1:**
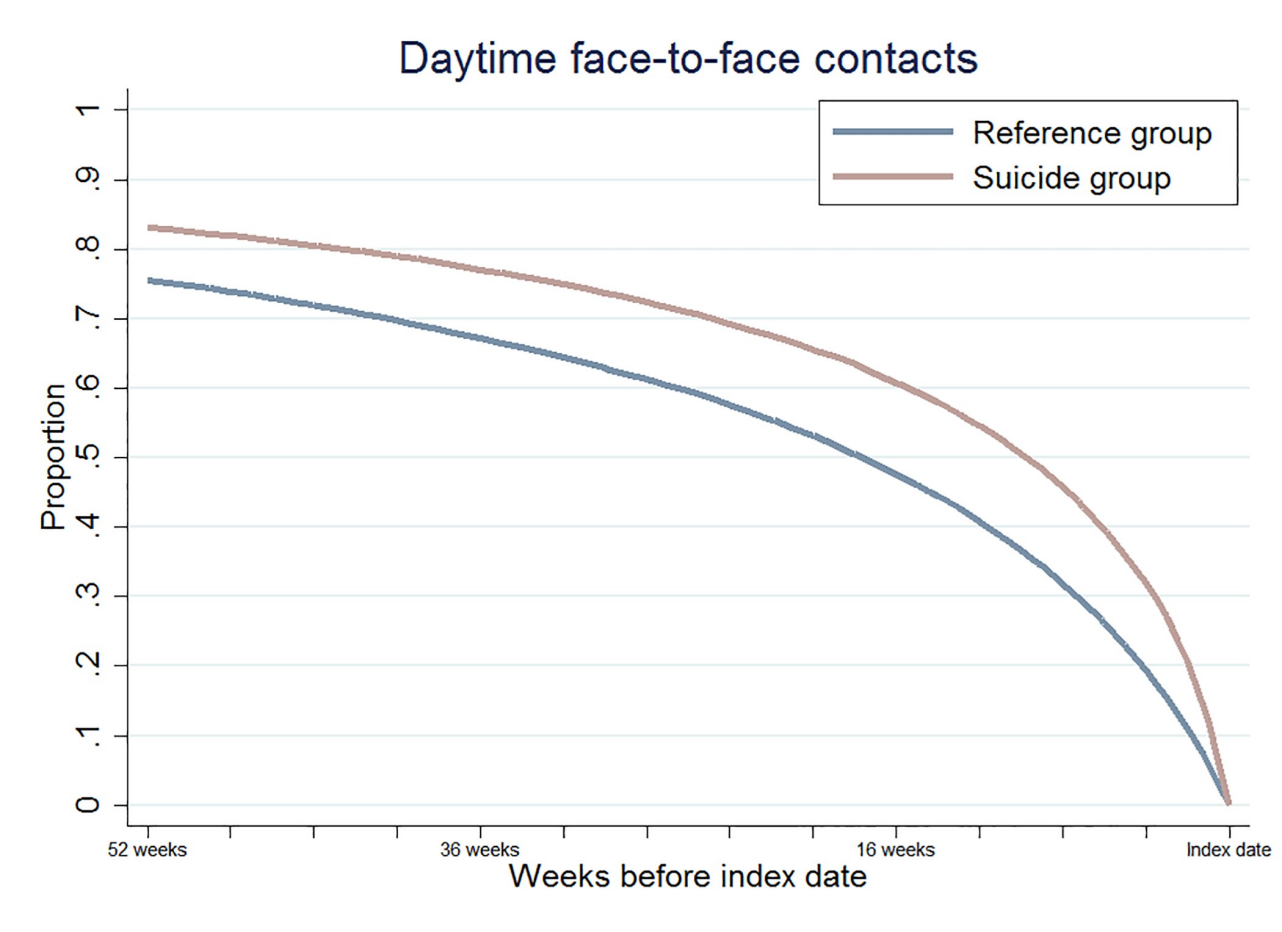
Backwards cumulative proportion of time of last daytime face-to-face contact before index date.

A higher percentage of the persons who died by suicide attended the GP when compared to the reference group, both in the year before index date (83% vs. 76%) and in the month before index date (32.0% vs. 19.4%) (Fig 1 and Table 2). This corresponds to a difference of 12.6 percentage points (PD: 12.6, 95% CI: (11.7; 13.5)) in model 1. The estimated difference was virtually unchanged when we adjusted for matching variables, cohabitation status, and educational level (PD: 12.5, 95% CI: (11.6; 13.4)). The estimated difference was slightly lower when we further adjusted for somatic diseases (PD: 12.0, 95% CI: (11.1; 12.9)).

**Table 2:** Proportion and proportion difference (PD) for daytime face-to-face contacts in the month before index date.

	Proportion (%) (95% CI)	PD (95% CI)		
		Model 1^a^	Model 2^b^	Model 3^c^
**Reference group**	19.4 (19.1; 19.7)	Ref	Ref	Ref
**Suicide group**	32.0 (31.1; 32.9)	12.6 (11.7; 13.5)	12.5 (11.6; 13.4)	12.0 (11.1; 12.9)

^a^Adjusted for age, sex, and calendar period.

^b^Adjusted for model 1 + socioeconomic status.

^c^Adjusted for model 2 + comorbid somatic diseases.

The subgroups with the largest unadjusted proportion of daytime face-to-face contacts in the month prior to suicide were suicide cases with two or more comorbid somatic diseases (42.2%), suicide cases who were recently bereaved (41.2%), and suicide cases older than 61 years of age (39.2%). All the variables in Table 3, except calendar time, tended to modify the difference; this was especially seen for cohabitation status and number of somatic comorbid diseases. For example, the PD was 9.8 percentage points for singles and 15.9 percentage points for recently bereaved individuals. Furthermore, the PD tended to decrease with increasing number of somatic comorbid diseases (Table 3).

**Table 3:** Proportion and proportion difference (PD) for daytime face-to-face contacts in the month before index date in subgroups.

	Reference group	Suicide group	
	Proportion (%) (95% CI)	Proportion (%) (95% CI)	PD (95% CI)^a^
**Sex**			
Male	17.7 (17.3; 18.1)	30.8 (29.8; 31.8)	12.7 (11.7; 13.8)
Female	23.9 (23.3; 24.6)	35.2 (33.5; 36.9)	10.1 (8.3; 11.8)
**Age (years)**			
-44	14.3 (13.8; 14.8)	25.7 (24.3; 27.1)	10.9 (9.4; 12.3)
45–61	17.3 (16.7; 17.8)	31.1 (29.6; 32.6)	13.3 (11.8,14.9)
62-	26.7 (26.1; 27.3)	39.2 (37.6; 40.7)	12.4 (10.7; 14.0)
**Year of index date**			
1997–2001	17.7 (17.2; 18.3)	30.6 (29.1; 32.1)	12.4 (10.8; 13.9)
2002–2007	20.2 (19.6; 20.8)	32.3 (30.8; 33.8)	11.6 (10.1; 13.2)
2008–2013	20.4 (19.8; 20.9)	33.1 (31.6; 34.6)	11.9 (10.4; 13.5)
**Number of comorbid somatic diseases**			
0	13.0 (12.7; 13.4)	25.8 (24.7; 27.0)	12.9 (11.7; 14.1)
1	22.8 (22.1; 23.6)	34.0 (32.1; 35.9)	11.5 (9.4; 13.5)
≥2	34.2 (33.3; 35.0)	42.2 (40.5; 44.0)	8.5 (6.5; 10.4)
**Educational level (years)**			
≤10	21.8 (21.2; 22.3)	33.0 (31.8; 34.3)	11.9 (10.5; 13.1)
11–15	18.2 (17.7; 18.7)	30.7 (29.4; 32.1)	11.8 (10.4; 13.2)
≥16	16.8 (16.1; 17.6)	32.1 (29.6; 34.6)	13.0 (10.5; 15.6)
**Cohabitation status**			
Single	19.2 (18.7; 19.8)	29.3 (28.1; 30.4)	9.8 (8.6; 11.1)
Recently bereaved	24.1 (19.7; 28.5)	41.2 (34.6; 47.9)	15.9 (8.5; 23.4)
Cohabiting	16.4 (15.5; 17.3)	29.2 (26.3; 32.0)	11.3 (8.3; 14.2)
Married	20.1 (19.6; 20.5)	36.3 (34.8; 37.8)	15.2 (13.7; 16.7)

^a^Mutually adjusted

When we excluded individuals who had been hospitalized within the last year before the index date, the estimated difference attenuated. We found that 10.6 percentage points more of those who died by suicide consulted the GP in the month before index date compared with the reference group (PD: 10.6, 95% CI: (9.4; 11.8)). The estimated difference did not change substantially when we excluded individuals listed with a GP who requested remuneration less than 35 times in the calendar year under study (PD: 12.2, 95% CI: (11.2; 13.2)).

**Fig 2:**
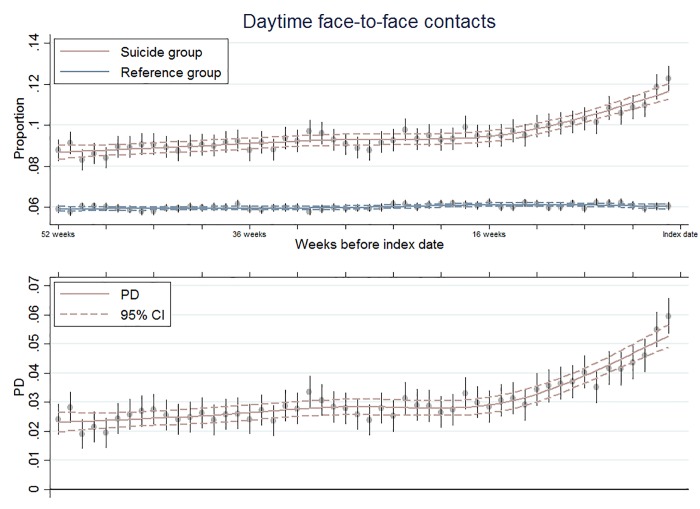
Raw proportion attending the GP and adjusted PD as a function of time. The PD estimates are adjusted for age, sex, calendar period, socioeconomic status, and comorbid somatic diseases.

We found that, throughout the year before the index date, a higher proportion of the suicide group than the reference group attended the GP on a weekly basis, even after we adjusted for all the variables in model 3. Furthermore, the PD tended to increase from approximately 4 months before the index date (Fig 2).

**Fig 3:**
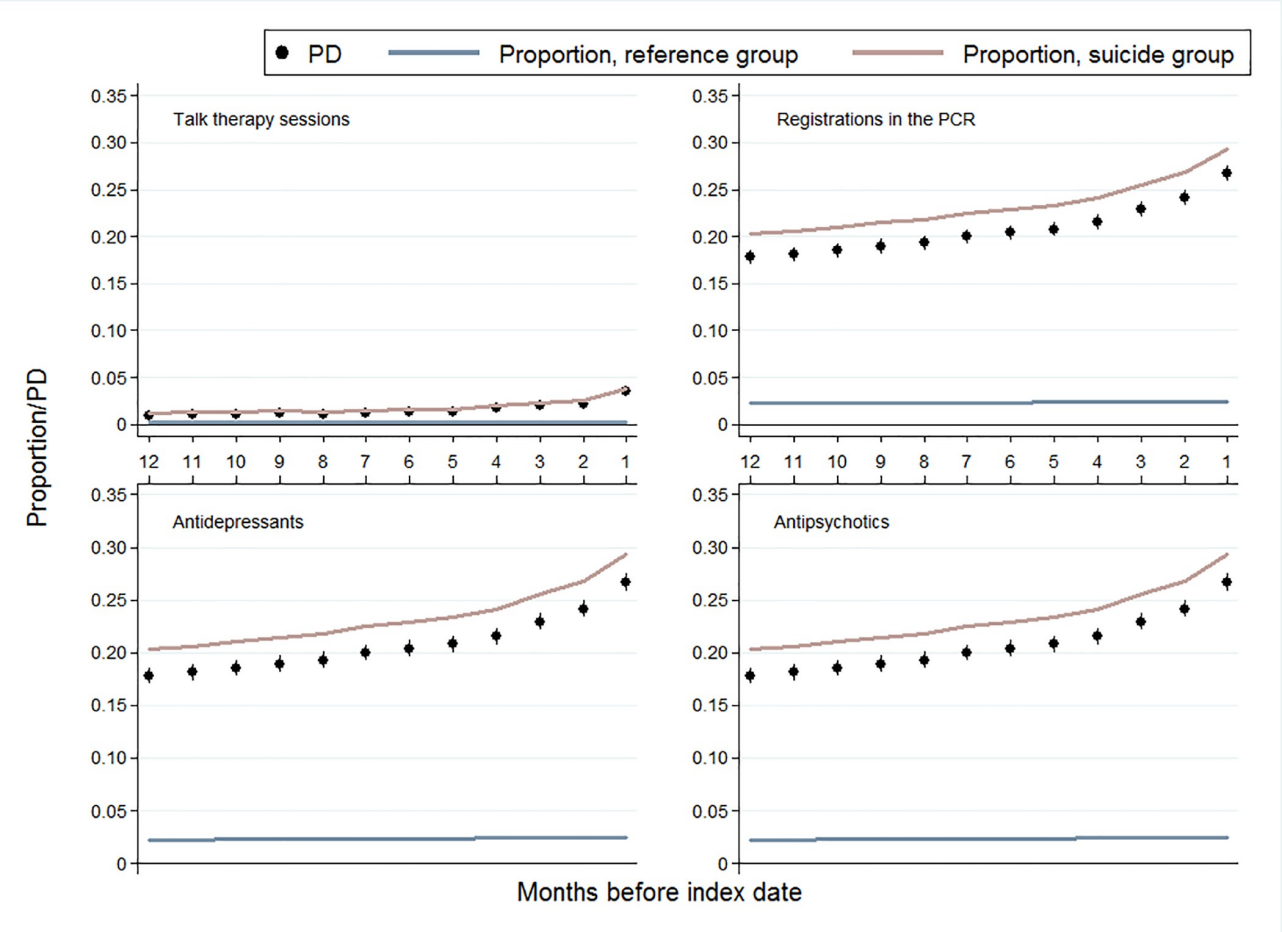
Talk therapy sessions, registrations in the PCR, and redeemed prescriptions for antidepressants, and antipsychotics stratified on months before suicide. All PD estimates are adjusted for age, sex, and calendar period.

**Fig 4:**
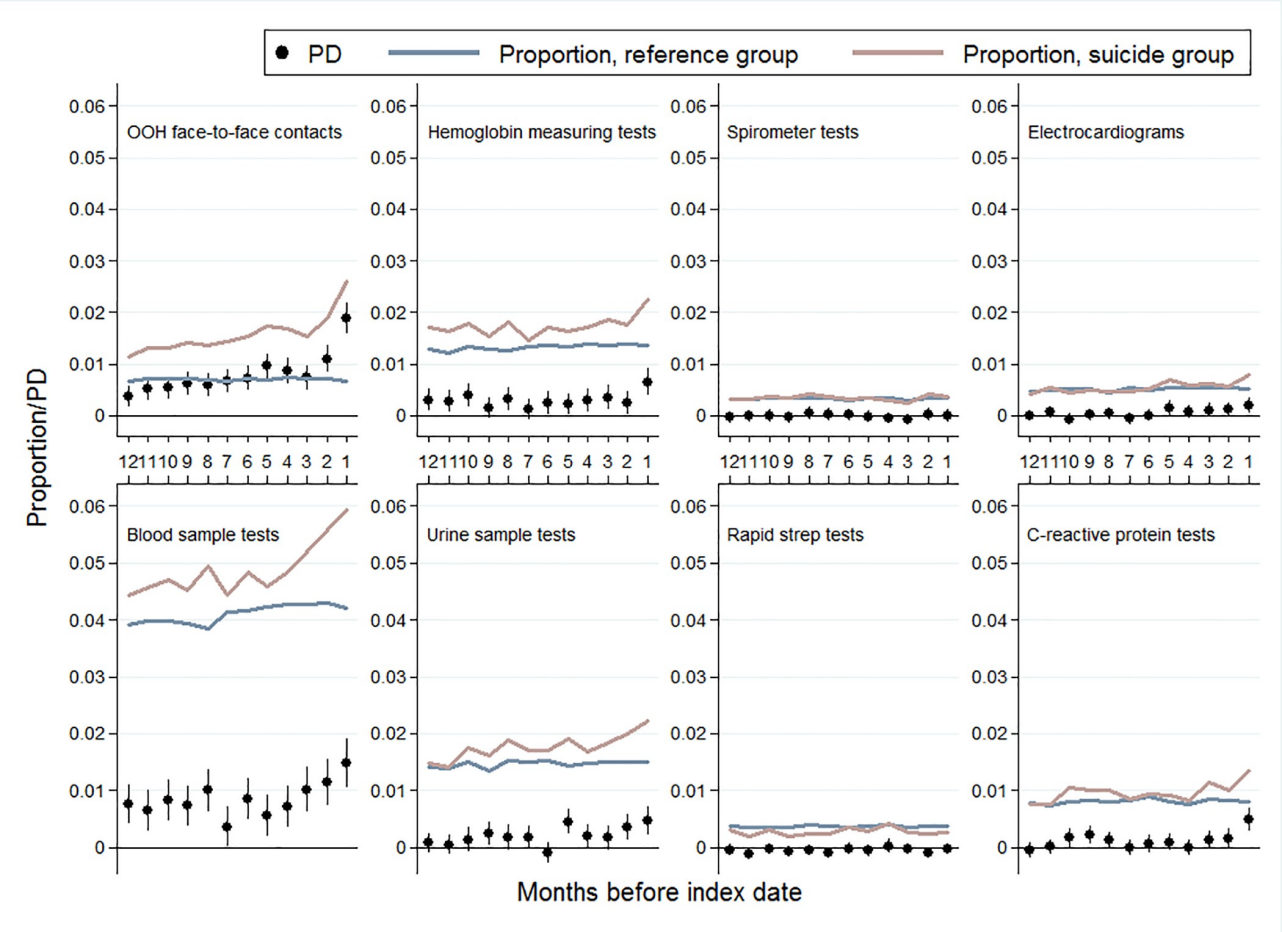
Out-of-hours face-to-face contacts and different types of contacts and tests taken in general practice stratified on months before suicide. All PD estimates are adjusted for age, sex, and calendar period.

Throughout the entire year before index date, a higher proportion in the suicide group had OOH contacts, talk therapy sessions, blood sample tests, psychiatric contacts, antidepressants treatment, and antipsychotics treatment compared to the reference group. Furthermore, the PDs for these outcomes increased during the last months before index date (Figs 3 and 4).

## Discussion

### Key results

In this nationwide matched comparative study ranging from 1997 through 2013, we identified more than 11,000 individuals who died by suicide. A total of 83% of these individuals attended a GP during the year prior to the suicide, the similar figure for the reference group was 76%. We found that, even a year before index date, a higher proportion had contacts related to mental health in the group who died by suicide compared to the reference group; this was seen both in primary care and in the psychiatric treatment system in secondary care. Likewise, a higher proportion of those dying by suicide were treated with antipsychotics and with antidepressants. In both daytime and OOH care, a greater proportion of the suicide cases had face-to-face contacts during the year before the suicide compared to the reference group, and an increased difference was seen in the last four months before the suicide.

### Strengths and limitations

To our knowledge, this is the largest study to date to investigate the frequency of health care utilization prior to completed suicide. A strength of this study is that we used data based on complete registrations of all suicides in Denmark during a 17-year period with practically no loss to follow-up. Additionally, we also had a matched comparison group. All data used were registered prospectively for administrative purposes, and we thus have no reason to suspect information bias or selection bias.

A limitation of using the NHISR is that we had no information about the reason for encounter with the GP. We only had information on the type of contact, e.g. talk therapy consultations or daytime face-to-face consultations. Another limitation of using the NHISR is that we did not have access to the actual treatment date, but only to the week in which the GP requested remuneration. Given that some GPs chose to request remuneration less frequently than once a week, we opted to test this in a sensitivity analysis. Yet, the results did not change when we excluded persons registered with one of these GPs.

A potential pitfall when analyzing attendance is that one has to be ‘at risk’ of attendance. One might suspect that a higher proportion in the suicide group than in the reference group are hospitalized during the year before index date. However, when we excluded individuals who had been hospitalized at some time point in the year before index date, the results did change a bit towards the null.

### Comparison with other studies

Our findings on the proportion of individuals attending the GP in the year and month before suicide are in line with the findings of previous studies and adds to the body of work. Ahmedani et al. [6] found that 83% had a health care visit in the year before suicide, and Pearson et al. [10] found that 91% attended the GP in the year prior to suicide. The latter study was, however, restricted to include only individuals who had first been in contact with a mental health institute. A Danish register-based study by Andersen et al. showed that a large proportion did have contacts to different health care providers in the year prior to suicide. This study was, however, geographically restricted to the county of Funen between 1991 and 1995 [12]. Unlike our study, none of the above mentioned studies included data from the background population. Hochman et al. included a reference group, but they restricted their study to males dying by suicide in the Israeli military. They found that 38.3% attended the GP in the month prior to dying by suicide vs. 33.8% in the control group [8].

### External validity

The results from this nationwide study are likely to be generalizable to other countries with a similar type of population demography and a free-of-charge health care system with the GP acting as gatekeeper to secondary care.

### Implications of findings

Previous studies have provided some support for GP-based suicide interventions [29], particularly with respect to identification of depression. Unützer et al. reported a lower rate of suicidal ideation among depressed older adults randomized to collaborative care in a primary care setting [30]. A recent meta-analysis by Milner et al. however found no clear statistical evidence of the effectiveness of suicide prevention delivered solely by GPs [31]. On the contrary a recent Danish register based study by Fenger-Grøn et al. found that the group of people who was recently bereaved had a significantly lower risk of e.g. suicide, if they were treated with talk therapy by their GP right after the loss [32]. The review by Milner et al. found conflicting results across the different studies on the effectiveness of GP-interventions for men and women.

Although attending the GP in general is conceived to be a beneficial act, it cannot be excluded that the patient may for example become suicidal after receiving unfavorable health news from their GP. Hence, some bidirectional effects might be assumed. We found that more persons from the suicide group attended the GP during the year before suicide, even after adjusting for socioeconomic characteristics and somatic comorbidity time-dependently, and that an increase in the difference was noted during the last four months; this suggest that the GPs do see people with suicidal ideation. The GPs should be encouraged to ask about suicidal thoughts when these seem to be present. Furthermore, it would be important to ensure that GPs have options for referrals to indicated interventions, such as psychosocial therapy [2,33].

## Conclusion

The findings from this study indicate that the GP potentially could play a role in the identification and intervention of susceptible individuals. Thus, it seems important to ensure that GPs have easy access to effective suicide prevention programs for patients at risk of suicide. However, it is important to note that the differences in GP utilization were small in our study and that only 32 percent of the individuals who died by suicide had seen a GP during the last month before dying. Combined, this evidence supports the notion that GP utilization is not a clinically relevant predictor of suicide in itself. Still, it would be recommendable to encourage persons at risk of suicide to contact the GP when having suicidal thoughts or plans.

## Supporting information

S1 Appendix (DOCX)
