# Supplementary material for: Frequency of health care utilization in the year prior to completed suicide: A Danish nationwide matched comparative study
Source: PLoS One. 2019 Mar 27;14(3):e0214605. doi: 10.1371/journal.pone.0214605 (PMC6436703; doi:10.1371/journal.pone.0214605)
Supplement: S1 Appendix — (DOCX) [file pone.0214605.s001.docx]

**Table A. Suicide cases identified in the Cause of Death Register**

| **Cause of death** | **Variable code** | **Diagnosis codes (ICD-10)** |
| --- | --- | --- |
| **Suicide** | c_liste14=11 | X60-X84, Y87 |

**Table B. Measurement type identified in the National Health Insurance Service Register**

| **Service type** | **Specialty code** | **Time code** | **Service code** |
| --- | --- | --- | --- |
| **Blood sample tests** | 80 | 0,1 | 2601,2101 |
| **Hemoglobin measuring tests** | 80 | 0,1 | 7108 |
| **Spirometer tests** | 80 | 0,1 | 7113,7121,7183 |
| **Electrocardiograpies** | 80 | 0,1 | 7156 |
| **Out-of-hours face-to-face contacts** | 80-89 | 8,9 | 0101 |
| **Talk therapy sessions** | 80 | 0,1 | 6101,4003,4021,4022,4023,  4024,4025,4026,4027,4050,  4063,4106,4247,4248,4249 |
| **Urine sample tests** | 80 | 0,1 | 7101 |
| **Rapid strep tests** | 80 | 0,1 | 7109 |
| **C-reactive protein tests** | 80 | 0,1 | 7120 |
| **Daytime face-to-face contacts** | 80 | 0,1 | 0101 |

**Table C. Measurement type identified in the Danish National Prescription Registry**

| **Prescription type** | **Drug code (ATC)** |
| --- | --- |
| **Antidepressants** | N06A |
| **Antipsychotics** | N05A |

**Table D. Educational level - From Statistics Denmark**

| ≤10 years |
| --- |
| 11-15 years |
| ≥16 years |

**Table E. Cohabitation status – From Statistics Denmark**

| Married |
| --- |
| Cohabiting |
| Single |
| Recently bereaved |

**Table F. Somatic diseases**

| **Category** | **Disease group** | **Coding definition** | **Diagnosis codes (ICD-10)** | **Diagnosis time frame** | **Drug codes (ATC)** | **Prescription time frame** |
| --- | --- | --- | --- | --- | --- | --- |
| **Circulatory system** | **Hypertension** | Diagnosis AND/OR prescriptions of antihypertensives, if not ischemic heart disease or heart failure (or kidney disease: only diuretics) | I10-I13, I15 | Ever | C02, C04, C07, C08, C09, C03 | Twice last year |
|  | **Dyslipidemia** | Diagnosis AND/OR drug prescription for lipid-lowering drugs if not ischemic heart disease. | E78 | Last two years | C10 | Twice last year |
|  | **Ischemic heart disease** | Diagnosis AND/OR prescription for antianginal drug | I20-I25 | Ever | C01DA | Twice last year |
|  | **Atrial fibrillation** | Diagnosis | I48 | Ever |  |  |
|  | **Heart failure** | Diagnosis | I50 | Ever |  |  |
|  | **Peripheral artery occlusive disease** | Diagnosis | I70-I74 | Ever |  |  |
|  | **Stroke** | Diagnosis | I60-I64, I69 | Ever |  |  |
| **Endocrine system** | **Diabetes mellitus** | Diagnosis AND/OR prescription of antidiabetics | E10-E14 | Ever | A10A, A10B | Twice last year |
|  | **Thyroid disorder** | Diagnosis AND/OR prescription of thyroid therapy drugs | E00-E05, E061-E069, E07 | Last two years | H03 | Twice last year |
|  | **Gout** | Diagnosis | E79, M10 | Ever |  |  |
| **Pulmonary system and allergy** | **Chronic pulmonary disease** | Prescription for obstructive airway disease drugs |  |  | R03 | Twice last year |
|  | **Allergy** | Prescription for non-sedative antihistamines AND/OR nasal antiallergics |  |  | R06AX, R06AE07, R06AE09, R01AC, R01AD | Twice last year |
| **Gastrointestinal system** | **Ulcer/chronic gastritis** | Diagnosis | K221, K25-K28, K293-K295 | Ever |  |  |
|  | **Chronic liver disease** | Diagnosis | B16-B19, K70-K74, K766, I85 | Ever |  |  |
|  | **Inflammatory bowel disease** | Diagnosis | K50-K51 | Ever |  |  |
|  | **Diverticular disease of intestine** | Diagnosis | K57 | Ever |  |  |
| **Urogenital system** | **Chronic kidney disease** | Diagnosis | N03, N11, N18-N19 | Ever |  |  |
|  | **Prostate disorders** | Diagnosis AND/OR prescription of prostate hyperplasia therapy drugs | N40 | Ever | C02CA, G04C | Twice last year |
| **Musculoskeletal system** | **Connective tissue disorders** | Diagnosis | M05-M06, M08-M09, M30-M36, D86 | Ever |  |  |
|  | **Osteoporosis** | Diagnosis AND/OR prescription for osteoporosis drugs | M80-M82 | Ever | M05B, G03XC01, H05AA | Twice last year |
|  | **Painful condition** | Repeated prescriptions of analgesics |  |  | N02A, N02BA51,N02BE, M01A, M02A | Four times last year |
| **Hematological system** | **HIV/AIDS** | Diagnosis | B20-B24 | Ever |  |  |
|  | **Anemias** | Diagnosis | D50-D53, D55-D59, D60-D61, D63-D64 | Last two years |  |  |
| **Cancers** | **Cancer** | Diagnosis | C00-C43, C45-C97 | Last five years |  |  |
| **Neurological system** | **Vision problem** | Diagnosis | H40, H25, H54 | Ever |  |  |
|  | **Hearing problem** | Diagnosis | H90-H91, H931 | Ever |  |  |
|  | **Migraine** | Diagnosis AND/OR prescription of specific anti-migraine drugs | G43 | Last two years | N02C | Twice last year |
|  | **Epilepsy** | Diagnosis AND prescription of anti-epileptics | G40-G47 | Ever | N03 | Twice last year |
|  | **Parkinson's disease** | Diagnosis | G20-G22 | Ever |  |  |
|  | **Multiple sclerosis** | Diagnosis | G35 | Ever |  |  |
|  | **Neuropathies** | Diagnosis | G50-G64 | Last two years |  |  |
